# Growlers

**Published:** 1876-12

**Authors:** 


### GROWLERS.

Some people seem to be in their natural element when they are grumbling, snapping, and snarling at every body and every thing; and, if the present does not afford them a text, they make drafts on future possibilities of ill. "Here, Bridget, it is almost daylight, Monday morning; to-morrow is Tuesday, and next day Wednesday, half the week gone, and no washing done yet." But every body does not feed on green persimmons. We could tell of a missionary who has been in the far West for twenty-one years. For a great part of that time he has lived among Indians, small pox, fevers, agues, and cholera, and, although not yet "fifty," looks prematurely old. For the last year or two his parishioners have paid him about a dollar a month. But does he rave and rail about the "ingratitude of republics?" Very far from it. He looks at the bright side of things, like a philosopher, or rather like a practical Christian. "I hardly know what it is to be under the weather, and think myself greatly blessed, even in earthly comforts. My appetite and digestion are good. I weigh about two hundred pounds. I have not had a chill in twenty years, until two months ago; am never confined to bed, except while asleep. I have done a good deal of hard work, and can do a good deal yet, for a kind Providence has prospered me."

One of the best pieces of philosophy we have heard for a long time, was uttered in a song at the rehearsal of Dr. Ward's beautiful opera last season. We do not recollect the words, but the sentiment was, that this world was bright or dark, as we take it ourselves—a world of sunshine to the light-hearted and the truly good, but to lower natures it was drear enough

### RESIGNATION.

With good health, a fine appetite, and a long purse, we rather think that most people could make this world one of flowers and smiles and sunshine. But to be old and sick and poor, and yet look upward through blinding tears of filial resignation, and say and feel "it is all right," that is only the Christian's feat; it is the miracle of religion.

